# Home Albumin Infusion Therapy, Another Alternative Treatment in Patients With Congenital Nephrotic Syndrome of the Finnish Type

**DOI:** 10.3389/fped.2020.614535

**Published:** 2021-01-14

**Authors:** Eugènia Serramontmany, Marina Muñoz, Aurora Fernández-Polo, María Morillo, Laura Gómez-Ganda, Carme Cañete-Ramírez, Gema Ariceta

**Affiliations:** ^1^Pharmacy Department, Vall d'Hebron University Hospital, Barcelona, Spain; ^2^Pediatric Nephrology Department, Vall d'Hebron University Hospital, Barcelona, Spain; ^3^Nursery Department, Vall d'Hebron University Hospital, Barcelona, Spain

**Keywords:** congenital nephrotic syndrome, finnish type, CNF, NPHS1, albumin, home albumin infusion therapy

## Abstract

**Background:** Congenital nephrotic syndrome of the Finnish type (CNF) is a rare, severe glomerular disease caused by mutations in the NPHS1 gene, which codes for nephrin. It is characterised by massive proteinuria and severe edoema. Progression to end-stage kidney failure occurs during early childhood and the only curative treatment is kidney transplantation. Nowadays, patients need aggressive medical treatment, which includes daily albumin infusions (for months) until they get clinical stability to receive transplant.

**Objective:** In our paediatric hospital, we implemented a multidisciplinary program for the home infusion of albumin with outpatient follow-up. The aim of the study was to assess the safety and efficacy of this program for the first four years of its implementation.

**Material and Methods:** Retrospective observational study of CNF paediatric patients treated with home albumin infusion therapy from March 2014 to July 2018 at a tertiary care paediatric hospital. Information on albumin administration was obtained from the electronic prescription assistance program and details on clinical and care-related variables from the hospital's electronic information systems.

**Results:** Four patients with CNF received albumin infusions for 18, 21, 22 months, and 3 years. The treatment was safe, and the complication rates were to be expected considering the severity of disease. Patients required a median of two hospital admissions a year (19 in total); 47% due to catheter-related complications, but there were just three catheter infections.

**Conclusions:** In our experience, home albumin infusion therapy is safe and effective and helps to improve children health and quality of life.

## Introduction

Nephrotic syndrome is the most common primary glomerular disease in the paediatric population. It is characterised by a glomerular lesion that leads to massive proteinuria (urine protein/creatinine ratio >2 mg/mg), resulting in hypoalbuminemia and generalised edoema. Nephrotic syndrome can be idiopathic or hereditary. Younger patients are more likely to have the hereditary form, and genetic diagnosis is recommended in all young patients with nephrotic syndrome who fail to respond to conventional treatment (1, 2). The most severe subgroup of nephrotic syndrome is congenital nephrotic syndrome (CNS), which is characterised by disease onset within the first 3 months of life, resistance to corticosteroids and immunosuppressive agents, and a very poor prognosis (3–5).

The prototype of severe hereditary nephrotic syndrome is CNS of the Finnish type (CNF) (OMIM: #256300), which, as its name suggests, is particularly prevalent in Finland, where it has an incidence of 1 case per 8,200 live births (4). However, CNF has been described in populations of different ethnic backgrounds around the world. It is an autosomal recessive disorder caused by loss-of-function mutations in the NPHS1 gene, leading up to alterations to the glomerular filtration barrier, responsible for the massive proteinuria typically seen in CNS (6, 7). Albumin is the main protein lost, but larger plasma proteins, such as immunoglobulins, may also be lost. Protein loss is correlated with hypoalbuminemia, severe malnutrition, hyperlipidemia, and an increased risk of thrombosis and infection (7).

Unlike idiopathic nephrotic syndrome, CNF is caused by a structural alteration. As such, it is resistant to corticosteroids and immunosupressants, and these treatments can even be harmful considering that patients are highly susceptible to infection (8).

As CNF is rare and challenging to manage, most patients are treated in specialised hospitals. Although prognosis has improved thanks to the introduction of early kidney transplantation and advances in dialysis techniques (9), patients still require an aggressive treatment approach to ensure that they survive until they attain an adequate weight and height for kidney transplantation (8).

In a study of 41 children diagnosed with CNF between 1953 and 1982, a team from the University of Minnesota led by Mahan et al. (10) found that all the children diagnosed before 1971 (before the introduction of early kidney transplantation) died. Those diagnosed later, however, showed 2-year survival rates of 82%.

As kidney transplantation is the only curative treatment for CNF, medical treatment is necessary to sustain good health and appropriate growth and development until the child is ready to undergo kidney transplantation (11). The goal of treatment thus is to control clinical manifestations to maintain clinical stability and prevent complications until the child attains a sufficient weight and height for transplantation.

The specific treatment aims are to control proteinuria and massive edoema through a high-calorie and high-protein diet and to prevent intercurrent infections and thromboembolic complications (3–12).

Various palliative treatments exist to control massive proteinuria and sustain clinical stability until the child is ready to undergo kidney transplantation.

The classic treatment for CNF, established by a group of Finnish experts two decades ago, is elective bilateral nephrectomy followed by dialysis until the moment of transplantation. This aggressive approach is justified by the presence of severe *NPHS1* mutations in the Finnish population (4, 5).

A more conservative approach, used at our hospital and other centres, consists of repeated intravenous albumin infusions associated with unilateral nephrectomy and/or treatment with antiproteinuric drugs to ensure clinical stability and normal glomerular filtration, thereby postponing the need for dialysis and transplantation and increasing the chances of success (11, 13–15).

This approach compensates for the massive loss of proteins and maintains intravascular oncotic pressure through daily intravenous infusions of albumin 20% (1 g/kg/day) in combination with furosemide (0.5 mg/kd/day) through a central venous catheter (CVC). In addition to a high-calorie and high-protein diet, patients are concomitantly prescribed drugs such as indomethacin (1–3 mg/kg/day) and angiotensin-converting enzyme inhibitors, to reduce glomerular filtration rate, and consequently proteinuria, and control severe edoema (15, 16).

Patients who remain clinically stable usually need to follow the above treatment for several months, i.e., until they are ready to undergo kidney transplantation. During this time, they typically need to be hospitalised or to make daily visits to the hospital for albumin infusions. This can have a major impact on the child's development and on family quality of life (5).

There have been growing reports of home infusion therapy programs being used in different fields that have proven to be both safe and effective. Examples are parenteral nutrition programs and antibiotic therapy programs for patients with cystic fibrosis (17, 18).

At our hospital we launched a multidisciplinary program for the home administration of intravenous albumin for children with CNF who only needed to be in hospital to receive this treatment. Apart from improving patient and family quality of life and favouring the children's personal and social development, the potential benefits of shortening hospital stays also included cost savings and a reduced risk of hospital-acquired infections.

The aim of this study was to assess the safety and efficacy of a home albumin infusion therapy program for paediatric patients with CNF from a tertiary care hospital during the first 4 years of the program and to evaluate associated morbidity.

## Method

The program was launched by the paediatric nephrology, pharmacy, and nursing departments in March 2014 to educate and train relatives and caregivers of patients with CNF in home albumin infusion therapy. The goal was to promote outpatient care and minimise hospital stays. The program targeted clinically stable patients whose only reason for being in hospital was to receive intravenous albumin and furosemide infusions. They were admitted to the program once their albumin infusion needs were reduced to a single daily infusion through a Broviac® CVC. This type of catheter was chosen to avoid the need for direct injections at home. Albumin was delivered by small, long-duration infusion pumps (Alaris™ GH Plus syringe pump from BD), provided by the hospital, that can be transported in dedicated backpacks to facilitate administration.

A multidisciplinary team formed by paediatric nephrologists, nursing staff, and pharmacists designed a series of health education and training sessions to teach the children's parents or caregivers to prepare and administer the albumin infusions in a sterile environment using the home infusion pumps.

Prior to discharge, parents also required to learn basic central line care skills to minimise risk of infection. There were also taught about warning signs (local signs in the area of the catheter and general signs such as edoema or fever) that they would need to watch for at home (Table 1). The nursing team evaluated the suitability of the home through a visit. In cases where the patient lived far from the hospital, the nursing team worked with the social worker of the area to assess the home.

**Table 1:** Interventions by the nursing in the home albumin infusion therapy program.

**Actions**	**Pre-discharge**	**Discharge**	**Weekly follow-up visits for first 6 months**	**Twice-monthly follow-up visits after first 6 months**
Primary caregiver training	Three training sessions on the knowledge, skills, and abilities needed for proper CVC^1^ care and albumin infusion	Evaluation of knowledge, skills, and abilities acquired via a checklist	- Consolidation of good home care practises - Clarification of doubts	- Consolidation of good home care practises- Clarification of doubts
*Broviac®* CVC monitoring	- Supervision of primary caregiver CVC handling - Training in technique for albumin infusion in a sterile environment	Verification of CVC securement and functioning	- CVC dressing - Entry-point dressing - Assessment of permeability reflux	- CVC dressing- Entry-point dressing- Assessment of permeability reflux
Blood tests			Capillary blood sampling	Capillary blood sampling
Anthropometric characteristics			Weight, height, waist circumference, and blood pressure	Weight, height, waist circumference, and blood pressure
Warning signs and actions	- Training in detection of local and systemic warning signs - Actions: differentiation between contact with assigned nurse (Monday to Friday 9:00–17:00) and emergency visits	- Review of knowledge acquired: detection of local and systemic warning signs- Consolidation of knowledge about when and how to act		
Contact person	Ward nurse and assigned nurse	- Assigned nurse from Monday to Friday 9:00–17:00- Emergency department	- Assigned nurse from Monday to Friday 9:00–17:00 - Emergency department	- Assigned nurse from Monday to Friday 9:00–17:00- Emergency department

1 *CVC, Central venous catheter*.

Once the home program was launched, the multidisciplinary team worked together to ensure that the follow-up visits with the different teams coincided to minimise the number of scheduled visits to the hospital.

The nursing staff also set up communication channels with the patients' primary care teams to optimise management and follow-up. Weekly visits with nursing staff and members of the paediatric nephrology team were also arranged to coincide. The aim of these visits was to cheque weight, height, waist circumference, blood pressure, CVC securement and dressings, and to adjust overall treatment as necessary. The protocol also envisaged a switch to twice-monthly visits after an initial period of 6 months (Table 1).

Medical supplies, ordered through an online system, were sent to the patient's home on a monthly basis, ensuring thus that the caregivers only needed to pick up the medication from the hospital pharmacy twice a month. These trips were designed to coincide with the follow-up visits.

This was a retrospective observational study of paediatric patients with genetically confirmed CNF treated with home albumin infusion therapy from March 2014 (launch of program) to July 2019 at a tertiary care paediatric university hospital.

Information was collected on biodemographic characteristics (sex, age, and weight); clinical data (date of diagnosis, infusion-related complications, and date of kidney transplantation); and care-related variables (number of CVC dressing changes, hospital admissions, and successive visits to the outpatient clinic to cheque the catheter).

Information on albumin administration and dosage was obtained from the Electronic Prescription Assistance Program used in our centre. Details on clinical and care-related variables were obtained from the hospital's electronic information systems.

## Results

Four patients, all boys, were included in the home albumin infusion therapy program. They were from four unrelated families, although three of them had a history of consanguinity. Two patients had been diagnosed with CNF during the first week of life and the other two were diagnosed between the ages of 1 and 2 months. The clinical diagnosis of CNF was confirmed genetically in all cases. Home albumin infusion therapy was started before the age of 4 months in all patients except the first boy recruited, who started when he was 6.5 months old (patient #1 in Table 2). The children were discharged from hospital when they required just a single daily infusion of albumin for <6 h. Median hospital stay during the children's first admission was 83 (range 43–192) days (Table 2).

**Table 2:** Clinical and demographic characteristics of patients.

	**Patient**			
	**1**	**2**	**3**	**4**
Age at diagnosis, days	2	71	23	7
Genetic diagnosis	c.1379G>A (p.Arg460Gln)	c.[1379G>A] + [2883G>A] (p.[Arg460Gln]+[Trp961^*^])	c.1135C>T(p.Arg379Trp)	c.3250_3251insG (p.Val1084Glyfs^*^12)
	Homozygosity	Compound heterozygosity	Homozygosity	Homozygosity
Post-diagnosis hospital stay, days	192 (6.5 months)	43 (1.5 months)	71 (2 months and 1 week)	83 (2.5 months)
Age at start of home albumin infusion therapy	6.5 months	4 months	2 months and 24 days	2 months and 23 days
Serum albumin at start of home albumin infusion therapy (g/dl)	2.7	2	2.18	2.1
Proteinuria prior to home albumin infusion therapy (mg/dl)^*^	3607.8	618	2,381	3,859
Time receiving home albumin infusion therapy	1 year and 6 months	3 years and 6 months	1 year and 10 months	1 year and 9 months
Age at time of kidney transplantation	2 years and 2 months	4 years and 10 months	2 years and 1 month	2 years
Dialysis before transplantation	No	No	No	No
Estimated glomerular filtration rate at time of kidney transplantation, mL/min (Schwartz equation)	15.55	19.5	6.82	14.57
Weight at the day of kidney transplantation, kg	10.4	18.9	11.1	11.5
Height at the day of kidney transplantation, cm	82.5	110.5	85	84
Weight percentile at the day of kidney transplantation	6.7	40	18.4	30.8
Height percentile at the day of kidney transplantation	2.3	44	18.4	11.5
First placement of *Broviac ^®^* CVC^1^, days	33	18	18	13
Number of CVC^1^	5	3	2	3
Current glomerular filtration rate, mL/min (Schwartz equation)	106.42	36.87	121.31	103.77
Current creatinine level, mg/dL	0.39	1.12	0.32	0.39
Mechanical complications of CVC^1^s	2	3	3	1
Catheter-associated infections	1	1	0	2
Other complications: pyelonephritis	0	1	0	1
Number of hospital admissions from diagnosis to transplantation	7	5	4	5
Number of admissions due to CVC complications	3	4	1	3

1 CVC, central venous catheter; NA, not applicable.

* *The value corresponds to the last determination prior to hospital discharge (1–2 months before discharge)*.

All the patients had a suitable family and home environment. Two of the families were Moroccan and two were Spanish. They were from very different sociocultural backgrounds, but this had no influence on their ability to acquire the necessary knowledge and skills covered in the training sessions. We also observed no association between lower sociocultural level and an increased rate of complications.

Albumin 20% was daily administered intravenously at a dose of 1 g/kg over 4 h (0, 1–0.5 ml/min). Furosemide (0.5 mg/kg/day) was administered as two doses: 50% halfway through the albumin infusion and 50% at the end. In all cases the drugs were delivered by a continuous-flow syringe pump through a *Broviac*® CVC (Table 2).

All the patients were also treated with high doses of indomethacin, and the antiproteinuric drug captopril.

The home infusion treatment goal (serum albumin levels of around 2 g/dL) was achieved in all cases and edoema was well-controlled. The median number of outpatient follow-up visits with the nursing staff and the paediatric nephrology team was 56 (range 48–96). Nursing advice was given by telephone on a median of 8 (range 2–14) occasions. Overall, 176 visits were made to the hospital pharmacy [median 44 (range 34–62) per patient] and 68 home medical supply deliveries were made [median 17 (range 6–36)].

A median of 3 (range 2–5) CVC changes per patient was required; these were due to partial dislodgement, breakage, or blockage (Table 2). Twenty one hospital admissions were required over the study period. This corresponds to a median of 2.5 admissions per patient per year; 52.4% of the admissions were due to CVC complications.

One of the admissions was due to infection of the CVC exit site by methicillin-resistant *Staphylococcus aureus* and required treatment with topical mupirocin and oral co-trimoxazole for 14 days. The same patient was admitted on two other occasions for catheter-related sepsis, caused by *Enterococcus cloacae* in one case (treated with meropenem) and methicillin-sensitive *S. aureus* in the other (treated with cloxacillin). One additional patient admitted due a respiratory infection episode and catheter exit site infection, was treated with oral cefadroxil as no germen was isolated. Of the remaining admissions, two were due to influenza B virus (in two vaccinated patients), two were due to acute pyelonephritis, and one was due to kidney failure resulting from acute gastroenteritis. One patient with hyporexia also required admission for percutaneous endoscopic gastrostomy (Table 2).

All patients, aged 24, 25, 26 months, and 4 years and 10 months, respectively, were discharged from the program when they underwent kidney transplantation and currently, they remain with functional grafts. In every case they received preemptive kidney transplantation without prior dialysis. The patient who was managed with medical treatment up to almost 5 years of age, had a less severe *NHPS1* mutation (Table 2).

## Discussion

Based on our experience, the home albumin infusion therapy program implemented at our hospital is safe and effective. The complication rates observed were no higher than expected considering the patients' condition, and none of the complications were related to the administration of albumin. None of the patients experienced life-threatening events and they progressed favourably up to the moment of kidney transplantation. The program resulted in shorter hospital stays. The patients were discharged home after the age of 4–6 months and remained in the program until they underwent transplantation (at ~2 years of age). Management by a multidisciplinary team formed by nursing staff, paediatric nephrologists, nutritionists, and pharmacists ensured the efficacy of treatment. One patient started home therapy later than the others (at 6.5 months of age) even though he had been diagnosed with CNF at just 2 days of age. This was because he was the first patient included and his parents needed to provide care to another child of theirs who had recently undergone kidney transplantation because of same disease.

Home infusion therapy programs for paediatric patients are increasingly being used in different fields, and based on the reports in the literature, they do not result in a significant increase in CVC-related complications. In addition, they have a favourable impact on patient and family quality of life as they reduce hospital admission times and enable a faster return to normal life (19).

Our results are consistent with those reported by a team at the North Children's Hospital in the UK, which has been running a paediatric home albumin infusion program for 25 years. Based on their experience with seven children with CNF, their program is safe and has not resulted in an increased rate of catheter-related complications (20).

We have already included four children since the launch of our program just 4 years ago. Like the UK program, our home albumin infusion therapy program is safe and has not resulted in any serious complications. Considering their risk profile (young age, chronic hypoalbuminemia, hypogammaglobulinemia, daily catheter use, etc.), patients with CNF can be expected to experience a high number of complications, as described by many specialist hospitals (8). In our series, there were very few infection-related complications (one entry-point infection and two cases of sepsis), attesting to the quality of nursing care provided and the success of the training sessions in which caregivers were taught how to administer the drug in a sterile environment. There were no thrombotic complications, although it should be noted that home infusion of alteplase has been found to significantly reduce the risk of intraluminal thrombosis (21). We, however, do not have any experience in this area.

Although we observed a high number of hospital admissions (*N* = 21) over the study period, again, this is to be expected given the severity of the patients' condition. Moreover, over half of the admissions were due to events unrelated to the albumin infusion program, such as viral infections and/or morbidity associated with the primary kidney disease. No life-threatening events occurred.

Further, with our conservative therapeutic approach, we avoided potential complications associated with early dialysis initiation children with CNF (5, 9), mainly in those patients who maintained native kidney renal function for extended period of time, as described (8).

It should be noted that implementation of the program increased the outpatient care burden for both the paediatric nephrologist and nursing teams, who had to closely monitor the patients, and the hospital pharmacists, who had to dispense the medications required and arrange for other supplies to be sent to the patients' homes.

This type of home programs is, especially, important in the current state of the SARS- CoV-2 pandemic since they allow reducing patient's or relative's exits from the home and hospital visits, thus reducing the possibility of contagion. In this scenario, recently another infant boy with CNF has successfully entered at the home albumin infusion therapy program in our centre.

### Limitations

The main limitation of this study is the small number of patients admitted to the program, but again, this is to be expected given the rarity of the disease. Nonetheless, the results obtained suggest that the program results in substantially shorter hospital stays and improved quality of life without compromising treatment efficacy and patient safety. Although we did not undertake a formal study of the impact of the program on quality of life, both the patients and their parents reacted very positively to being able to continue treatment at home.

A second limitation of our study is that we were unable to compare the economic impact of the home program with in-hospital care and treatment, as we did not have a control group.

### Conclusions

Based on our experience, a paediatric home albumin infusion therapy program run under the supervision of an expert team is both safe and effective. In our opinion, this treatment modality should be offered to all paediatric patients with CNF who have reached the requirement of a single daily dose of albumin within the framework of a structured caregiver training program. We observed no significant increase in catheter-related complications, adverse drug effects, or albumin infusion reactions.

Our home albumin infusion therapy program allows patients and their families to continue treatment in the comfort of their home. It favours growth and development (and may even improve general health), reduces the need for long hospital stays, and offers an opportunity to optimise healthcare resource utilisation. Finally, it can reduce the psychological and social sequelae associated with this disease in early childhood.

## Data Availability Statement

The original contributions presented in the study are included in the article/supplementary materials, further inquiries can be directed to the corresponding author.

## Author Contributions

ES, AF-P, MMo, LG-G, and CC-R: data collection. ES, MMu, AF-P, LG-G, and CC-R: data analysis. ES, MMu, and AF-P: manuscript writing. AF-P and GA: review. All authors contributed to the article and approved the submitted version.

## Conflict of Interest

The authors declare that the research was conducted in the absence of any commercial or financial relationships that could be construed as a potential conflict of interest.
